# Generational Factors Associated with SARS-CoV-2 Vaccine Completion for Americans of Mexican Decent Living along the United States–Mexico Border Region

**DOI:** 10.3390/vaccines12101137

**Published:** 2024-10-03

**Authors:** Francisco Soto, Argentina E. Servin, Davey M. Smith, Fatima Muñoz, Jeannette L. Aldous, Jamila K. Stockman, Daniel Ramirez, Britt Skaathun

**Affiliations:** 1 Division of Infectious Diseases and Global Public Health, School of Medicine, University of California, La Jolla, San Diego, CA 92093, USA; fsoto@health.ucsd.edu (F.S.); d13smith@health.ucsd.edu (D.M.S.); jstockman@health.ucsd.edu (J.K.S.); 2 UC San Diego School of Medicine, La Jolla, San Diego, CA 92093, USA; argentinanoelle@gmail.com; 3 San Ysidro Health, San Diego, CA 92173, USA; fatima.munoz@syhealth.org (F.M.); jlaldous@syhealth.org (J.L.A.); daniel.ramirez@syhealth.org (D.R.)

**Keywords:** COVID-19, vaccine hesitancy, generational factors, vaccine series completion, vaccine uptake, SARS-CoV-2, Latino, African American, public health, health disparities, age generations

## Abstract

**Background:** SARS-CoV-2 vaccine uptake variation remains a significant barrier to overcoming the spread of COVID-19. Individual beliefs/attitudes about the SARS-CoV-2 vaccine vary significantly across generations due to personal experiences, access to accurate information, education levels, political beliefs, and trust in healthcare systems. **Methods:** This analysis used data from the baseline visit of *Project 2VIDA!*, a cohort of Americans of Mexican descent (AoDM) and African American individuals (n = 1052) in San Diego, CA, along the U.S.–Mexico border region. The survey assessed sociodemographics, healthcare access, socioeconomic factors, and trust in public health information/SARS-CoV-2 prevention. We conducted a logistic regression involving AoDM individuals to identify generational factors associated with completing the SARS-CoV-2 vaccine series. **Results:** The results of the logistic regression analysis revealed that Generation X (OR = 0.52, 95% CI = 0.33–0.82), Millennials (OR = 0.24, 95% CI = 0.14–0.41), and Generation Z (OR = 0.10, 95% CI = 0.05–0.22) were less likely to complete the SARS-CoV-2 vaccine series when compared to Baby Boomers and the Silent Generation. **Conclusions:** Participants with a history of SARS-CoV-2 testing and trust in the SARS-CoV-2 vaccine were significantly more likely to complete the SARS-CoV-2 vaccine series. Efforts to address vaccine series completion should be tailored to the specific concerns and motivations of different age groups.

## 1. Introduction

SARS-CoV-2 vaccine uptake variation across demographic groups remains a public health barrier to overcoming the spread of the virus in the United States (U.S.) and globally [1]. According to world data tracked by the Centers for Disease Control and Prevention (CDC), only 32.7% of people in low-income countries have received their first dose [1]. In 2024, CDC reported that only 20.7% of the U.S. population had received an Updated (Bivalent) Booster Dose [1]. Information tracked by the California CDC in 2023 has revealed that more than 90% of individuals in age categories ranging from 18 to 65+ years have received their first dose of the COVID-19 vaccine [1]. Individual beliefs and attitudes about the SARS-CoV-2 vaccine can vary significantly within each generation due to personal experiences, socioeconomic status, education levels, political beliefs, trust in the healthcare system, and vaccine hesitancy [2,3,4,5]. In the initial phases of SARS-CoV-2 vaccination in the U.S., older age groups were prioritized because older individuals were more susceptible to severe outcomes leading to high mortality rates [6]. As eligibility expanded to younger age groups, efforts were made to promote awareness and accessibility for other demographics [5,7]. Generational cohorts such as the Silent Generation (born 1925–1945), Baby Boomers (born 1946–1964), Generation X (born 1965–1979), Millennials (born 1980–1994), and Generation Z (born 1995–2012) may exhibit different attitudes and behaviors regarding vaccination [5]. Given the potential for vaccines to reduce disease severity and transmission, it is critical to understand how to improve SARS-CoV-2 vaccine uptake and series completion. However, the majority of the existing research on SARS-CoV-2 vaccine hesitancy has predominantly centered on factors like race/ethnicity, gender, educational attainment, income status, and health insurance coverage [8,9,10].

For instance, disparities exist in SARS-CoV-2 vaccine uptake among racial and ethnic minorities, with lower vaccination rates observed among African Americans and Latinos despite racial/ethnic minorities being at elevated risk for COVID-19-related mortality [11]. Systematic reviews have reported that amongst ethnic and racial minorities, Hispanic Americans are 30% more hesitant to receive the SARS-CoV-2 vaccine [12]. Previous studies have reported that amongst Latino populations living in the U.S., Mexican Americans experience higher rates of vaccine hesitancy and increased mortality rates from COVID-19 when compared to other Latino subgroups [7,12,13,14,15]. Although research has been conducted on vaccine hesitancy in Latino demographics, it has failed to focus on the variety of intersecting identities within the Latino community [7,12,14,15,16,17]. This creates barriers to accessing health resources and remains a challenge in overcoming vaccine hesitancy [7,12,14,15,16,17]. Recent research on vaccine hesitancy has recommended that Latino identity not be treated as a monolithic factor when evaluating SARS-CoV-2 vaccine uptake in the United States, as nuances within this group, such as age, primary language spoken, gender, and country of origin are also important [13,18,19]. Thus, the proposed study seeks to fill the gap in the literature by focusing on Americans of Mexican descent living along the U.S.–Mexico border and determining whether generational factors are associated with SARS-CoV-2 vaccine series completion among this group [5].

## 2. Materials and Methods

### 2.1. Study Design and Data Source

We used the baseline survey of *Project 2VIDA!* (Project 2VIDA! SARS-CoV-2 Vaccine Intervention Delivery for Adults in Southern California. R01MD016872; PI: Servin. ClinicalTrail.gov Identifier: NCT05022472) a federally funded multicentric cluster randomized controlled trial centered on addressing COVID-19 vaccine hesitancy among Latino and African American individuals (n = 1052) in San Diego, CA, along the U.S.–Mexico border region [20]. The survey assessed sociodemographic characteristics, vaccination hesitancy, vaccine series completion, characteristics associated with social marginalization (e.g., food insecurity, substance abuse, engagement in commercial sex work, intimate partner violence), medical history, access to and utilization of healthcare, trust in medical and public health information and COVID-19 prevention behaviors. As published elsewhere, inclusion criteria included (a) age of 16 years or older, (b) identifying as Latino or African American, (c) being assigned male or female at birth, (d) being a resident of one of the six communities selected for this study (National City, Lincoln Park, Logan Heights, Valencia Park, Chula Vista or San Ysidro), (e) literate in English or Spanish, (f) no known history of severe allergic reactions to any components of the vaccine, (g) no history of immune disease, (h) not currently pregnant, (i) no plans to move from the area in the following 30 days, (j) able to provide voluntary informed consent and (k) able to provide complete contact information. It is important to note that the San Diego/Tijuana border is a unique area, with the two cities being only 15 min apart. Many U.S. citizens reside at least part-time in Tijuana due to factors such as cost of living and proximity to family members who do not have the ability to reside in the U.S. In 2020, the Centers for Disease Control and Prevention estimated that ‘799,000 U.S. citizens resided in Mexico, often living in border communities and traveling between the countries for work, healthcare, and family obligations’ [21]. We focused on individuals who identified as being of Mexican descent (who themselves, their parents, grandparents, or later generations have ties to Mexico) (n = 642) and conducted a logistic regression to identify generational factors associated with COVID-19 vaccine series completion.

#### 2.1.1. Setting

The study targeted regions in San Diego County with the highest reported COVID-19 cases, focusing on National City, Logan Heights, Lincoln Park, Valencia Park, Chula Vista, and San Ysidro [20].

#### 2.1.2. Recruitment

Various recruitment strategies were performed, including seeking participants through collaborations with community partners, utilizing social media platforms, and widespread distribution of flyers in easily accessible locations like grocery stores, community-based organizations, parks, and local eateries [20].

#### 2.1.3. Inclusive Representation

Special attention was paid to ensuring diverse representation within the study sample, aligning with San Diego County’s racial and ethnic demographics [20]. Specifically, efforts were made to ensure that at least 40% of participants identified as women [20].

#### 2.1.4. Inclusion Criteria

Inclusion criteria were carefully crafted to encompass individuals aged ‘16 and above’ who identified as Latinx and/or African American, resided within the targeted communities, and were proficient in either English or Spanish [20].

#### 2.1.5. Ethical Considerations

Certain exclusion criteria, such as pregnancy and the inability to provide consent, were established to uphold ethical standards and participant safety. Participants were assured that declining to participate would not affect their access to essential healthcare services or other benefits [20]. The study was approved by the Human Research Protections Program (HRPP) at the University of California, San Diego and San Ysidro Health’s ad hoc IRB committee. Additionally, all participants provided voluntary consent prior to participating in the study. The manuscript does not contain any identifying personal data.

#### 2.1.6. Randomization and Control Measures

Participating communities were assigned to either the intervention or control group using a computer-generated random sequence [20]. Control sites underwent training to ensure consistency in process evaluation and quality assurance protocols across all study sites [20].

#### 2.1.7. Intervention Framework

The 2VIDA! intervention was designed based on a robust framework informed by the National Institute on Minority Health and Health Disparities and principles of Community-Based Participatory Research (CBPR) [20]. It sought to mitigate health disparities by focusing on multiple facets, including COVID-19 awareness and education, linkage to medical and supportive services, community outreach, and vaccine distribution [20].

#### 2.1.8. Phase-Based Intervention

Phase 1 of the intervention centered on educational outreach and community engagement, aiming to bolster COVID-19 awareness and preparedness [20]. This involved distributing culturally sensitive educational materials, conducting outreach activities, and establishing a COVID-19 resource center for individual education and service linkage [20]. Phase 2 focused on administering SARS-CoV-2 vaccines through community pop-up clinics in targeted areas, addressing access barriers in African American and Latino communities [20].

#### 2.1.9. Data Collection and Evaluation

Baseline and follow-up surveys were conducted to comprehensively assess factors influencing vaccine access, acceptance, and uptake [20]. These surveys covered a range of topics, including sociodemographic characteristics, healthcare utilization, vaccination history, perceptions of the vaccine, and trust in health agencies [20]. Research staff remained available to offer support throughout the survey process, ensuring participant comprehension and engagement [20].

#### 2.1.10. Compensation

Participants received a USD 20 VISA gift card upon completing the baseline survey and were eligible for an additional USD 20 VISA gift card upon completing the follow-up survey. Incentives were provided as a token of appreciation for participants’ time and contribution to the research efforts, enhancing motivation and engagement without exerting undue influence on their decision-making processes.

### 2.2. Measures

The outcome, vaccine series completion, was assessed based on the completion of the series as specified by the CDC’s Advisory Committee on Immunization Practices (CDC, 2019) and the California Immunization Registry (CAIR) [1]. We assessed the association between demographics and other characteristics with ‘SARS-CoV-2 vaccine series completion’ (How many SARS-CoV-2 vaccinations have you received?) [20]. The dependent variable for this study, ‘SARS-CoV-2 vaccine series completion’, is a binary variable (yes vs. no). The dependent variable for this study, ‘SARS-CoV-2 vaccine series completion’, was measured as ‘no’, including ‘none’, ‘one dose’, ‘two doses’, and ‘yes’ including ‘three doses’.

The independent variables included in the logistic regression model were ‘sex’, ‘generational age’, ‘education’, ‘COVID-19 food challenges’, ‘COVID-19 test history’, and ‘trust in SARS-CoV-2 vaccine’. Sex was classified as assigned male and female at birth. Education survey responses included ‘Less than high school’, ‘high school graduate or GED’, and ‘post high school graduate’. Generational age was classified chronologically according to the following categories: Generation Z (born 1995–2012, 11–28 years old at time of survey), Millennials (born 1980–1994, 29–43 years old at time of survey), Generation X (born 1965–1979, 44–58 years old at time of survey), Baby Boomers (born 1946–1964, 59–77 years old at time of survey), and the Silent Generation (born 1925–1945, 78–98 years old at time of survey) [5]. The generational age categories Baby Boomers and Silent Generation were included in one category because research has shown that the Baby Boomer and Silent Generation age categories have reported higher use and trust in the healthcare system due to the time that they have had to develop this trust [5]. COVID-19 food insecurity was assessed by asking, ‘As a result of the COVID-19 pandemic in March 2020, did you or your family experience…not enough money to pay for food?’ and it was included as a binary variable (yes vs. no). COVID-19 test history was assessed based on the question ‘Have you ever taken a COVID-19 test?’ through a binary variable (yes vs. no). Trust in the SARS-CoV-2 vaccine was also assessed with the question, ‘Do you trust that a vaccine will help protect you against COVID-19?’ with a binary variable response (yes vs. no).

### 2.3. Analysis

Distribution percentages were calculated for demographics including sex, generational age, education, COVID-19 food insecurity, trust in the SARS-CoV-2 vaccine and COVID-19 testing history. For generational age, Generation Z has an age time frame of 11–28 years. For this analysis, we focused on people of 18 years of age or older per participant eligibility. Bivariate analyses were conducted to access associations between the independent variables and COVID-19 vaccine series completion via chi-square tests of independence. Seven independent variables with significance with a *p*-value of less or slightly less than 0.001 were identified and incorporated into the logistic regression model. As outlined in previous studies, a multivariable logistic regression model was used to adjust for variables that demonstrated a statistically significant bivariable association and known factors impacting vaccine completion [22,23,24].

## 3. Results

### 3.1. Descriptive Statistics

Over half of the sample (60.7%) analyzed identified as assigned male at birth and 39.3% as assigned female at birth. For ‘generational age’, the distribution was as follows: ‘Generation Z’ (9.3%), ‘Millennials’ (16.8%), ‘Generation X’ (30.7%), and ‘Baby Boomers/Silent Generation’ (43.1%). The generational age categories Baby Boomers and Silent Generation were included in one category because research has shown that Baby Boomers and the Silent Generation age categories have been reported to have higher use and trust in the healthcare system due to the time that they have had to develop this trust [5]. Approximately half of the sample (54.4%) reported having less than a high school education. Approximately 30.4% of the participants reported experiencing food insecurity due to the COVID-19 pandemic. Approximately 80% of participants reported ever testing for COVID-19. Trust in the SARS-CoV-2 vaccine is consistent with this health behavior, since 10.6% of participants reported no trust in the COVID-19 vaccine. The dependent variable, ‘SARS-CoV-2 vaccine series completion’, had a distribution of ‘no’ (34.3%) and ‘yes’ (65.7%) (Table 1).

### 3.2. Bivariate Analyses

In bivariate analyses, gender, generational age, education, economic hardship associated with the pandemic, COVID-19 testing history, and trust in the COVID-19 vaccine were all associated with vaccine series completion (Table 2).

### 3.3. Multivariate Logistic Regression Analysis

Results of the logistic regression analysis revealed that Generation X (OR = 0.52, 95% CI = 0.33–0.82), Millennials (OR = 0.24, 95% CI = 0.14–0.41), and Generation Z (OR = 0.10, 95% CI = 0.05–0.22) were less likely to complete the SARS-CoV-2 vaccine series when compared to the Baby Boomers and Silent generation age categories. Participants with a history of SARS-CoV-2 testing (OR = 3.60, 95% CI = 2.27–5.70) and trust in the SARS-CoV-2 vaccine (OR = 2.32, 95% CI = 1.28–4.21), were significantly more likely to complete the COVID-19 vaccine series. Amongst those who answered yes or no to having a COVID-19 testing history, those who have a testing history are more likely to complete the COVID-19 vaccine series (OR = 3.60, 95% CI: 2.27–5.70, *p* < 0.001). Those who answered that they had trust in the SARS-CoV-2 Vaccine were more likely to complete the COVID-19 vaccine series (OR = 2.32, 95% CI: 1.28–4.21, *p* = 0.006) (Table 3).

## 4. Discussion

The results from this study highlight that among this sample, Generation X, Millennials and Generation Z were less likely to complete the SARS-CoV-2 vaccine series compared to the Baby Boomers and Silent Generation age categories. Further, our findings indicate that individuals with a history of SARS-CoV-2 testing and trust in the SARS-CoV-2 vaccine were significantly more likely to complete the SARS-CoV-2 vaccine series. The implications of these findings are discussed below.

Previous research analyzing race and class as predictors of SARS-CoV-2 vaccine hesitancy found that racial and ethnic minorities have lower trust in the SARS-CoV-2 vaccine due to factors such as racism, less access to health resources, and historical bio-medical abuse [19]. The unfortunate history of unethical medical experimentation in the U.S., such as the Tuskegee Syphilis Study, has led to mistrust towards public health initiatives among marginalized groups [25]. Structural racism also impacts health inequities, where disparities in healthcare access, quality of care, and discriminatory practices fuel skepticism about the intentions and safety of government-led medical interventions [26].

Communities of color also often experience greater exposure to misinformation regarding vaccines, compounded by a lack of culturally competent health communication from public health officials, which further diminishes vaccine confidence [27]. Studies by López-Cevallos et al. (2015) emphasize that interventions engaging Latino communities should incorporate community values, language, and trusted community leaders to bridge gaps in trust. Utilizing community health workers (‘promotores de salud’) or influential figures within these generations to promote vaccine benefits, share facts about safety, and debunk myths is recommended [28]. Our findings on ‘trust’ being significantly associated with vaccine completion suggest that Latinos in this region may increase SARS-CoV-2 vaccine uptake when administered by their primary provider when that provider is trusted. These findings emphasize the importance of culturally competent care and public health messaging.

Research has shown that Generation Z has a higher COVID-19 incidence compared to Millennials, Generation X, Baby Boomers, and the Silent Generation [29]. Our findings suggest that there is a need to develop age-appropriate prevention strategies and interventions that improve vaccine uptake and completion, specifically within generational ages Generation X, Millennials, and Generation Z. Generation Z (currently aged 18–29 years) are the least likely to complete the vaccine series. This, coupled with the high incidence of COVID-19 in this population and the high probability of community transmission of COVID-19 from Generation Z to older generations [30], highlights the importance of developing Generation Z age-appropriate COVID-19 prevention strategies and interventions, specifically for Latino subcultures. Younger generations, especially Generation Z and Millennials, are more likely to respond to digital campaigns and social media outreach. A study by Latkin et al. (2021) supports the idea of ‘social influence campaigns’, where individuals are encouraged to share their positive vaccination experiences online. Testimonials from people within their own social networks and influencers from their communities can help counteract misinformation and encourage vaccine series completion [31].

Previous literature on generational age and vaccine uptake is consistent with our findings, even when stratified into the Latino subpopulation, who are consistent in their positive attitude towards vaccine uptake interventions and efforts [5]. Compared to other generations, Baby Boomers and the Silent Generation have had more time to establish a positive relationship with healthcare resources and providers [5]. Previous cross-sectional analyses on vaccine hesitancy have revealed that Generation Z tends to have a positive attitude towards vaccines [5]. Although our analysis suggests that those belonging to Generation Z were less likely to complete the vaccine series, this potential positive attitude should be leveraged to develop age-appropriate vaccine intervention information and dissemination.

Millennials and Generation Z are often more susceptible to misinformation due to the high volume of information they consume via social media. A systematic review by Wang et al. (2021) demonstrated that tailored interventions that directly address misinformation about vaccine safety and efficacy are essential in boosting vaccine confidence among younger populations. These interventions should include credible influencers, provide access to fact-checked resources, and engage with users through interactive formats like webinars or live question-and-answer sessions on social media [32].

Finally, we would like to highlight the importance of community-based interventions that integrate educational campaigns with accessible vaccination services, such as the present study, for increasing vaccination among Americans of Mexican descent. According to a study by Fisher et al. (2020), community-driven approaches, such as partnering with local churches, schools, and grocery stores in Mexican American neighborhoods, can help reduce vaccine hesitancy by making vaccines more convenient and building trust through familiar community institutions [33]. Programs should address language barriers by providing materials in Spanish and involving bilingual staff to answer questions. Hosting vaccination drives in convenient locations with minimal bureaucratic barriers will further increase completion rates among these younger generations.

This study on the completion of the SARS-CoV-2 vaccine series across various generations has two main limitations. One limitation is the exclusion of individuals below 18 years of age from Generation Z, which may impact the accuracy of our findings for this group. The second limitation is that utilization of self-reported data for variables such as ‘trust’ may introduce response biases, providing a modest incentive may introduce bias, and the study’s cross-sectional design limits our capacity to establish causality. Moreover, our specific emphasis on Americans of Mexican descent may restrict the generalizability of our results to other ethnic or racial groups, as different cultural norms and experiences may influence medical decision-making differently.

## 5. Conclusions

Our results reveal that age and trust in the SARS-CoV-2 vaccine among this sample of Latino individuals are significant factors contributing to vaccine series completion. Efforts to address vaccine series completion should be tailored to the specific concerns and motivations of generational age groups, specifically Generation Z (20–29 years), due to reported high incidence and the potential consequence of non-vaccination on facilitating community transmission among the Baby Boomer and Silent Generation age categories (59–98 years). Future prevention interventions should aim to provide clear, accurate, and accessible information about the safety and efficacy of SARS-CoV-2 vaccines, prioritizing those with high incidence risk. Centering on Americans of Mexican descent and the Generation Z generational age category with regard to vaccine uptake will not only have an effect on incidence within Generation Z, but will reduce community transmission to other generations as well.

## Figures and Tables

**Table 1:** Distribution of participant characteristics (n = 642).

Study Variables	Percent Mean	Sample Size
**Gender**		
Male	60.7%	390
Female	39.3%	252
**Generational Age**		
Generation Z	9.3%	60
Millennials	16.8%	108
Generation X	30.7%	197
Baby Boomers and Silent	43.1%	277
**Education**		
Less than a high school education	54.4%	349
Graduated from high school (preparatoria) or has a GED	19.8%	127
Post high school education	25.9%	166
**COVID-19 Challenges: Not enough money to pay for food**		
No	69.6%	447
Yes	30.4%	195
**COVID-19 test history**		
No	20.1%	129
Yes	79.6%	511
**COVID-19 Challenges: Not enough money to pay for rent**		
No	62.5%	401
Yes	37.5%	241
**Trust in COVID-19 Vaccine**		
No	10.6%	68
Yes	89.4%	574
**COVID-19 Series Completion**		
No	34.3%	218
Yes	65.7%	417

**Table 2:** Bivariate associations between COVID-19 vaccine completion and participant characteristics (n = 642).

Characteristic	Vaccine Series Completion		*p*-Value (Chi-Square Test)
	Yes n (%)	No N (%)	
**Gender**			0.015
Female	267 (69.4%)	118(30.6%)	
Male	150 (60%)	100 (40%)	
**Generational Age**			<0.001
Generation Z	12 (20.3%)	47(79.7%)	
Millennials	46 (43.4%)	60 (56.6%)	
Generation X	135 (68.9%)	61 (31.1%)	
Baby Boomers and Silent	224 (81.8%)	50 (18.2%)	
**Education**			<0.001
Less than high school	260 (76.9%)	78 (23.1%)	
Graduated from high school (preparatoria) or has GED	62 (49.6%)	63 (50.4%)	
Post high school education	90 (54.9%)	74 (45.1%)	
**COVID-19 Challenges**			
**Not enough money to pay for food**			0.003
No	274 (62%)	168 (38%)	
Yes	143 (74.1%)	50 (25.9%)	
**COVID-19 test**			<0.001
No	53 (41.4%)	75 (58.6%)	
Yes	363 (71.9%)	142 (28.1%)	
**Not enough money to pay for rent**			0.522
No	257(64%)	140 (37.5%)	
Yes	160 (67.2%)	78 (32.8%)	
**Trust in COVID-19 Vaccine**			<0.001
No	27 (40.3%)	40 (59.7%)	
Yes	390 (68.7%)	179 (31.3%)	

**Table 3:** Multivariable logistic regression analysis: factors associated with COVID-19 vaccine completion (n = 620).

Study Variables	Odds Ratio	95% Confidence Interval (95% CI)	*p*-Value
**Gender**			
Ref: Male			
Female	1.19	0.80–1.75	0.395
**Generational Age**			
Ref: Baby Boomers and Silent Generation			
Generation Z	0.102	0.047–0.221	<0.001
Millennials	0.236	0.137–0.405	<0.001
Generation X	0.518	0.326–0.823	0.005
**Education**			
Ref: Post high school education			
Less than high school	1.50	0.923–2.418	0.103
Graduated from high school (preparatoria) or has GED	0.80	0.466–1.387	0.433
**COVID-19 Challenges**			
**Not enough money to pay for food**	1.43	0.928–2.212	0.105
**COVID-19 testing history**	3.60	2.274–5.700	<0.001
**Trust in COVID-19 Vaccine**	2.32	1.280–4.207	0.006
**Constant**			0.091

## Data Availability

The data from this manuscript is available upon reasonable request from the corresponding author.
